# Effect of a conditional cash transfer programme on leprosy treatment adherence and cure in patients from the nationwide 100 Million Brazilian Cohort: a quasi-experimental study

**DOI:** 10.1016/S1473-3099(19)30624-3

**Published:** 2020-05

**Authors:** Julia M Pescarini, Elizabeth Williamson, Joilda S Nery, Anna Ramond, Maria Yury Ichihara, Rosemeire L Fiaccone, Maria Lucia F Penna, Liam Smeeth, Laura C Rodrigues, Gerson O Penna, Elizabeth B Brickley, Mauricio L Barreto

**Affiliations:** a Centre for Data and Knowledge Integration for Health (CIDACS), Instituto Gonçalo Moniz, Fundação Oswaldo Cruz (FIOCRUZ), Salvador, Brazil; b Department of Medical Statistics, London School of Hygiene & Tropical Medicine, London, UK; c Department of Non-communicable Disease Epidemiology, London School of Hygiene & Tropical Medicine, London, UK; d Department of Infectious Disease Epidemiology, London School of Hygiene & Tropical Medicine, London, UK; e Health Data Research, London, UK; f Instituto de Saúde Coletiva, Universidade Federal da Bahia, Salvador, Brazil; g Instituto de Matemática e Estatística, Universidade Federal da Bahia, Salvador, Brazil; h Universidade Federal Fluminense, Instituto de Saúde da Comunidade, Niterói, Brazil; i Núcleo de Medicina Tropical, Universidade de Brasília, Escola FIOCRUZ de Governo Fundação Oswaldo Crus Brasília, Brazil

## Abstract

**Background:**

Indirect financial costs and barriers to health-care access might contribute to leprosy treatment non-adherence. We estimated the association of the Brazilian conditional cash transfer programme, the Programa Bolsa Família (PBF), on leprosy treatment adherence and cure in patients in Brazil.

**Methods:**

In this quasi-experimental study, we linked baseline demographic and socioeconomic information for individuals who entered the 100 Million Brazilian Cohort between Jan 1, 2007, and Dec 31, 2014, with the PBF payroll database and the Information System for Notifiable Diseases, which includes nationwide leprosy registries. Individuals were eligible for inclusion if they had a household member older than 15 years and had not received PBF aid or been diagnosed with leprosy before entering the 100 Million Brazilian Cohort; they were excluded if they were partial receivers of PBF benefits, had missing data, or had a monthly per-capita income greater than BRL200 (US$50). Individuals who were PBF beneficiaries before leprosy diagnosis were matched to those who were not beneficiaries through propensity-score matching (1:1) with replacement on the basis of baseline covariates, including sex, age, race or ethnicity, education, work, income, place of residence, and household characteristics. We used logistic regression to assess the average treatment effect on the treated of receipt of PBF benefits on leprosy treatment adherence (six or more multidrug therapy doses for paucibacillary cases or 12 or more doses for multibacillary cases) and cure in individuals of all ages. We stratified our analysis according to operational disease classification (paucibacillary or multibacillary). We also did a subgroup analysis of paediatric leprosy restricted to children aged up to 15 years.

**Findings:**

We included 11 456 new leprosy cases, of whom 8750 (76·3%) had received PBF before diagnosis and 2706 (23·6%) had not. Overall, 9508 (83·0%) patients adhered to treatment and 10 077 (88·0%) were cured. After propensity score matching, receiving PBF before diagnosis was associated with adherence to treatment (OR 1·22, 95% CI 1·01–1·48) and cure (1·26, 1·01–1·58). PBF receipt did not significantly improve treatment adherence (1·37, 0·98–1·91) or cure (1·12, 0·75–1·67) in patients with paucibacillary leprosy. For patients with multibacillary disease, PBF beneficiaries had better treatment adherence (1·37, 1·08–1·74) and cure (1·43, 1·09–1·90) than non-beneficiaries. In the propensity score-matched analysis in 2654 children younger than 15 years with leprosy, PBF exposure was not associated with leprosy treatment adherence (1·55, 0·89–2·68) or cure (1·57, 0·83–2·97).

**Interpretation:**

Our results suggest that being a beneficiary of the PBF, which facilitates cash transfers and improved access to health care, is associated with greater leprosy multidrug therapy adherence and cure in multibacillary cases. These results are especially relevant for patients with multibacillary disease, who are treated for a longer period and have lower cure rates than those with paucibacillary disease.

**Funding:**

CONFAP/ESRC/MRC/BBSRC/CNPq/FAPDF–Doenças Negligenciadas, the UK Medical Research Council, the Wellcome Trust, and Coordenação de Aperfeiçoamento de Pessoal de Nível Superior–Brazil (CAPES).

## Introduction

Leprosy, also known as Hansen's disease, is a neglected tropical disease that affects more than 200 000 individuals worldwide annually and is a leading infectious cause of permanent physical disability.^1^, ^2^ As the burden of leprosy-associated disability can be mitigated through timely detection and treatment, WHO has supplied free multidrug therapy to health systems in high-burden countries since 1995.^1^ WHO recommends a 6-month treatment regimen for patients with paucibacillary leprosy (ie, those with five lesions or fewer), comprising daily doses of dapsone.^1^ For patients with multibacillary leprosy (ie, those with more than five lesions or a positive slit-skin smear), the recommendation comprises a 12-month treatment regimen with daily combined doses of dapsone and clofazimine. For both operational classifications of the disease, the daily treatments are accompanied by once-monthly doses of rifampicin administered under supervision.^1^ Not completing the prescribed treatment regimens for leprosy can contribute to ongoing transmission, stigmatised disabilities, and antimicrobial resistance.^3^

**Research in context**

**Evidence before this study**

In low-income and middle-income countries, social protection policies have been associated with better tuberculosis treatment outcomes. To investigate the available evidence of the effect of such programmes on leprosy, we searched PubMed and Embase for studies published in any language between Jan 1, 1990, and April 18, 2019, containing the following terms: (“financial support” [MeSH Term] OR “cash transfer program” OR “cash transfer” OR “public assistance” [MeSH Term] OR “social protection” OR “monetary incentive” OR “social programs” OR “food assistance” OR “food program” OR “social policies” OR “social policy” OR “safety nets” OR “in-kind transfers”) AND (“leprosy” OR leprosy[MeSH Terms] OR “Hansen”). We found 628 records and identified only two relevant studies that evaluated any associations between receiving social protection benefits and leprosy incidence, prevalence, related disabilites, and treatment outcomes. Both studies had an ecological design and evaluated the association between the conditional cash transfer programme Programa Bolsa Família (PBF) and leprosy incidence in Brazil at the municipality level. One study focused on the effect of the programme on children younger than 15 years. Both studies found an approximately 15% reduced leprosy incidence in municipalities with high coverage of PBF, but neither evaluated its effect on treatment adherence or cure.

**Added value of this study**

To our knowledge, this is the first study to evaluate the association between a policy aiming to reduce poverty and leprosy treatment outcomes. By linking nationwide data from individuals applying for social programmes in Brazil and analysing data from more than 11 000 new leprosy cases, our study has unprecedented statistical power to study leprosy treatment outcomes. Using a causal inference framework of analysis and propensity score methods to control for socioeconomic and demographic characteristics, we found that receiving PBF benefits was associated with improved adherence to multidrug therapy treatment and increased cure rates. Our results show the public health potential of using linked administrative datasets to study the effect of social policies on the outcomes of rare diseases, including neglected tropical diseases, and provide new evidence of a potential beneficial effect of cash transfer programmes on leprosy treatment and control.

**Implications of all the available evidence**

To policymakers, our study contributes to the evidence base that programmes that mitigate poverty might bolster leprosy control and should be considered essential tools for helping countries to achieve the goals outlined in the WHO Global Leprosy Strategy 2016–2020. Although further research is needed to identify the specific mechanisms by which participation in cash transfer programmes improves adherence to treatment and cure in patients with leprosy, this study indicates that cash transer or in-kind social assistance programmes have the potential to improve leprosy treatment outcomes.

Individuals living in poor socioeconomic conditions in low-income and middle-income countries are disproportionately affected by leprosy.^4^ In Brazil, the country with the second-highest number of leprosy cases worldwide after India, individuals with poor socioeconomic status and unfavourable living conditions have a substantially increased risk of being newly diagnosed with leprosy.^5^ Additionally, adverse socioeconomic factors might influence adherence and completion of leprosy treatment. A 2009 population-based survey in northern Brazil found a positive association between non-adherence and dropout from leprosy treatment and the poverty-related characteristics of low familial income, fewer rooms per household, and migration.^3^ Furthermore, a 2016 population-based survey in China reported that a leprosy diagnosis can incur large financial costs (ie, up to 38% of annual household income) and lead to job insecurity, which could exacerbate the risks of treatment dropout.^6^ Additionally, a 2013 systematic review found that health-service engagement with patients with leprosy has been associated with increased retention in care in India, Brazil, and the Philippines.^7^

**Figure 1:**
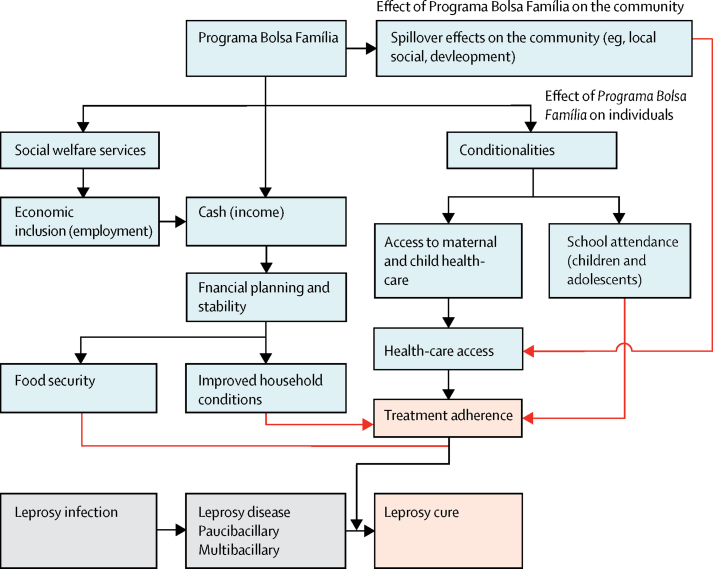
Hypothetical model of the potential pathways by which the Programa Bolsa Família might affect leprosy treatment adherence and cure Black arrows indicate factors known to be affected directly (ie, individual socioeconomic conditions), whereas red arrows indicate factors that might affect leprosy treatment adherence and cure.

Worldwide, conditional cash transfer programmes, including the Brazilian Government's social welfare Programa Bolsa Família (family allowance programme; PBF) implemented in 2004, have benefitted recipient families by reducing poverty,^8^ improving health-care use,^9^, ^10^ and increasing cure rates for chronic infections such as tuberculosis.^11^, ^12^, ^13^, ^14^, ^15^ Although increased PBF coverage has been associated with reduced leprosy incidence in Brazilian municipalities,^16^, ^17^ the influence of conditional cash transfers on leprosy treatment patterns is unknown. We hypothesised that participation in the PBF has the potential to increase leprosy treatment adherence and cure by reducing the financial consequences of a leprosy diagnosis and strengthening the interaction of patients with health services (figure 1).^10^, ^18^ Using prospective data from more than 10 000 patients with leprosy in the 100 Million Brazilian Cohort, we investigated the association of the PBF with indicators of retention in care. Specifically, we evaluated whether patients with leprosy who began receiving cash transfers from the PBF before their diagnosis were more likely to complete the prescribed multidrug therapy regimen and be cured than their counterparts who did not benefit from the PBF programme.^1^

## Methods

### Study design and participants

In this quasi-experimental study, we included newly diagnosed patients with leprosy detected among individuals who entered the 100 Million Brazilian Cohort between Jan 1, 2007, and Dec 31, 2014, the time period for which data on leprosy were available. We considered individuals as exposed to the PBF intervention if they had started receiving PBF benefits before their leprosy diagnosis and continued receiving benefits during the entire duration of treatment. Individuals who never received PBF aid over the duration of multidrug therapy treatment were considered to be unexposed to the PBF intervention.

Newly diagnosed patients were included in our analysis if they had a household member older than 15 years (to exclude children registered separately from any adult family members), and had not received PBF, nor had been diagnosed with leprosy before entering the 100 Million Brazilian Cohort. After selecting patients who met these conditions, we excluded individuals who were partial receivers of PBF benefits (ie, who stopped receiving PBF benefits either before diagnosis or during treatment), had missing data for the outcome variables and baseline familial sociodemographic covariates, or had a monthly per-capita income greater than BRL200 (US$50; ie, the highest quartile of incomes in the 100 Million Brazilian Cohort) at baseline.

This study was done under the Declaration of Helsinki and the Brazilian and UK research regulations and was approved by the three research ethics committee of the University of Brasília (Brasília, Brazil; 1.822.125), Instituto Gonçalo Muniz (Salvador, Brazil; 1.612.302), and London School of Hygiene & Tropical Medicine (London, UK; 10580-1). The 100 Million Brazilian Cohort has waived the need for informed consent, as it has been built through the linkage of administrative databases.

### Data sources

Data on newly diagnosed patients with leprosy from the 100 Million Brazilian Cohort were identified through the linkage of the baseline of the 100 Million Brazilian Cohort and the Information System for Notifiable Diseases (SINAN) of the Brazilian Ministry of Health. Information on PBF receipt was obtained through the linkage of the baseline of the 100 Million Brazilian Cohort and the PBF payroll database. The full linked cohort was provided by the Centre for Data and Knowledge Integration for Health (CIDACS; Salvador, Brazil).^19^, ^20^

The baseline of the 100 Million Brazilian Cohort was built from the initial application of families and their family members for social assistance programmes in Brazil through the registration with the national administrative database Cadastro Único para Programas Sociais (CadÚnico) from Jan 1, 2001, to Dec 31, 2015.^21^ Individuals aged 16 years and older can subscribe to CadÚnico, and on registration, individual (ie, sex, age, race or ethnicity, education, and work status) and familial (ie, familial income, household density, and housing characteristics) sociodemographic information is collected.^21^

Families registered in CadÚnico are eligible to receive PBF funding if they are extremely poor (ie, receiving ≤BRL60 [approximately $15·0] monthly per capita in 2007–08 and ≤BRL70 [$17·5] monthly per capita in 2009–14) or poor (ie, ≤BRL120 [$30·0] monthly per capita in 2007–08 and ≤BRL140 [$35·0] monthly per capita in 2009–14) and have children (≤17 years old), pregnant women, or breastfeeding women in the household.^21^ Eligible families receive a monthly cash transfer conditional on school attendance of all children, health monitoring and vaccination for children aged up to 6 years, and attendance of prenatal and postnatal care for women. Social assistants monitor families receiving PBF benefits to ensure compliance, and the income benefit is suspended only after a minimum of 2 years of non-compliance.^21^ We extracted information from the PBF payroll database about the date of the first and last payments transferred to families from Jan 1, 2004, to Dec 31, 2015.

SINAN is a decentralised surveillance system, operated in partnership with health-care professionals in all health-care facilities, that monitors the incidence of notifiable diseases and collects data to inform the provisioning of health services and resources. When a leprosy diagnosis is made, health professionals collect sociodemographic (ie, sex, age, race or ethnicity, education, and work status) and clinical (ie, date of diagnosis and case type [new or relapsed]) information, operational classification (paucibacillary or multibacillary disease), and grade of disabilities at diagnosis (0, 1, and 2). Multidrug therapy data are recorded during and after treatment and include the dates of treatment initiation and last visit, the number of doses taken, and the treatment outcome (ie, cure, transferred away, dropout, or death). We extracted sociodemographic, clinical, and treatment data on all new leprosy cases that were notified to SINAN between Jan 1, 2007, and Dec 31, 2014.

### Dataset linkage

Data extraction and linkage procedures were done at CIDACS. The 100 Million Brazilian Cohort (baseline 2001–15) and PBF payroll database (2004–15) were linked using exact deterministic linkage through each individual's Number of Social Identification, a unique identifier. The Cohort's baseline and SINAN (available leprosy data 2007–14) were deterministically linked using the CIDACS-RL tool, an open-source record linkage tool registered in GitHub, which generates a similarity score on the basis of five individual identifiers: name, date of birth, sex, name of mother, and the municipality of residence.^22^ Linkage accuracy was evaluated by two independent researchers who manually reviewed 10 000 randomly selected linked pairs from different score strata to verify the proportion of true and false matched pairs in each strata.^23^ Random selection was done using a computer-generated randomised sample using R software. Disagreement was resolved by a third senior researcher. Sensitivity and specificity were assessed for various threshold of the similarity score. The best performing score threshold that was selected for use in our analysis (≥0·92) achieved a specificity of 0·89 (95% CI 0·88–0·90) and sensitivity of 0·91 (95% CI 0·90–0·92; data not shown).

### Outcomes

Outcomes of interest were leprosy treatment adherence and cure. According to WHO guidelines, leprosy treatment is completed when patients finish a 6-month multidrug therapy regimen in 9 months or fewer for paucibacillary leprosy or a 12-month regimen in 18 months or fewer for multibacillary leprosy.^1^ In Brazil, leprosy cure is recorded by health professionals in SINAN when these guidelines are met, but additional multidrug therapy doses can be prescribed in the absence of clinical improvements on the clinician's discretion.^24^ We defined leprosy treatment adherence as achieving the prescribed number of multidrug therapy doses (ie, six or more doses for paucibacillary disease and 12 or more doses for multibacillary disease) and leprosy cure as recorded by health professionals.

### Statistical analysis

We used propensity score matching to estimate the effect of PBF aid receipt on leprosy treatment outcomes. We used logistic regression to estimate the propensity scores of receiving PBF aid, considering all of the following baseline demographic and socioeconomic covariates collected before receipt of aid and leprosy diagnosis: sex, age, race or ethnicity, education, work, per-capita income, household density, geographic region, area of residence (urban *vs* rural), housing ownership, housing construction material, electricity, water supply, and sewage and waste disposal. We considered the education and work characteristics of individuals younger than 18 years to be represented by the oldest member in the family (as a proxy for the family head). For individuals enrolling into the 100 Million Brazilian Cohort after Aug 31, 2009, we divided the per-capita income by 1·167 to account for the same rate of change in the eligibility criteria for the PBF. Variables were selected a priori for inclusion in the fully adjusted model on the basis of potential confounders for the association between PBF aid and leprosy treatment outcomes (ie, adjustment for all variables was made simultaneously).

We matched beneficiaries and non-beneficiaries (1:1) using nearest-neighbour matching with replacement and a caliper of 0·05.^25^ Missing data in individual sociodemographic covariates, which we assumed were potentially stronger confounders of the association between PBF aid and the individually measured outcomes than the covariates assessed at the familial level, were included as a category. We assessed the balance in the distribution of covariates before and after matching using standardised mean difference (a difference of <0·1 after matching was considered to indicate a good balance). Propensity-score estimation and matching were done for the overall sample and for paucibacillary and multibacillary cases separately. We estimated the average treatment effect on the treated (ATT) by calculating the odds ratio in each matched dataset using logistic regression, with further adjustment for income as a continuous variable, using robust SEs clustered by individual to account for matching with replacement.^26^ Additionally, as paediatric leprosy diagnoses indicate relatively high endemicity and active transmission,^27^ we did a subgroup analysis in children who were younger than 15 years at the time of leprosy diagnosis, as this age threshold is used by the Brazilian Ministry of Health and WHO as an indicator of leprosy control. These analyses were done in all individuals who met the study inclusion criteria.

We did sensitivity analyses related to our analytical approach and the definition of cure. First, we used the inverse probability of treatment weighting (IPTW) to estimate the ATT of PBF on leprosy treatment outcomes. For this analysis, the effect of PBF aid on treatment adherence and cure was estimated using logistic regression, weighting non-beneficiaries with the formula propensity score / (1–propensity score) and beneficiaries as 1, with further adjustment for income. This analysis was done for the overall population and for individuals younger than 15 years. Second, to investigate the robustness of our conclusions regarding the way in which missing data in studied covariates were handled, we did a complete case analysis (ie, restricting the analysis to individuals without missing data for any of the covariates included in the propensity score). Third, to test if the results are robust regarding the definition of cure, we excluded individuals recorded as cured in SINAN who had not completed the minimum number of multidrug therapy doses for each operational classification form of the disease and re-estimated the effect in the overall population, using both propensity-score matching and IPTW with the propensity score. All analyses were done using STATA version 15.0 and R version 3.5.2.

### Role of the funding source

The funders of the study had no role in study design, data collection, data analysis, data interpretation, or writing of the report. The corresponding author had full access to the data and had final responsibility for the decision to submit for publication.

## Results

**Figure 2:**
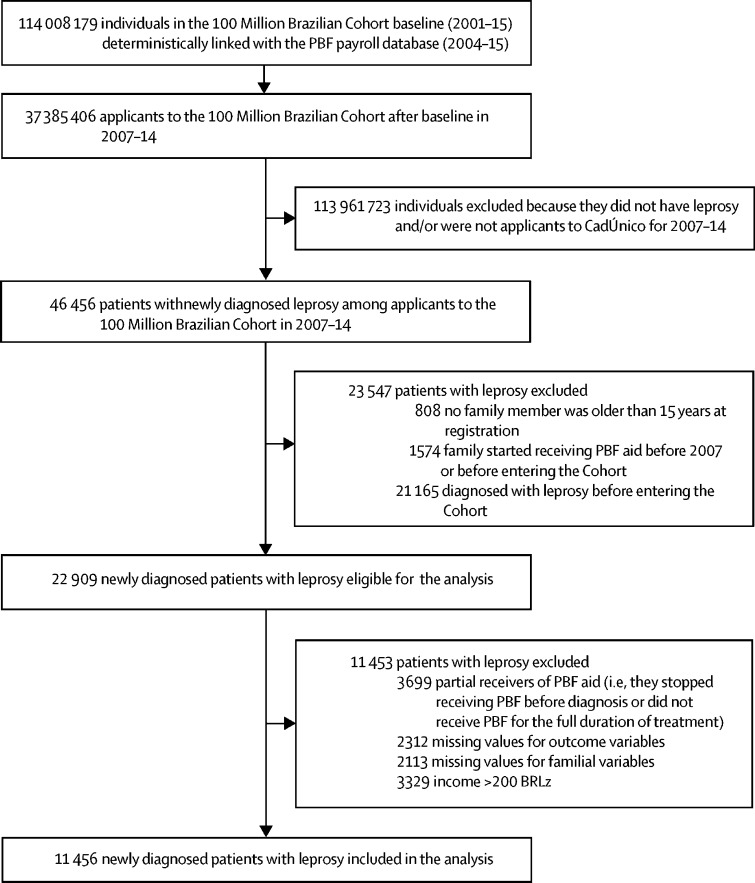
Flow diagram CadÚnico=Cadastro Único para Programas Sociais. PBF=Programa Bolsa Família.

From 2001 to 2015, the 100 Million Brazilian Cohort baseline linked with the PBF payroll database included socioeconomic and PBF information on 114 008 179 individuals (figure 2). When these individuals were linked with the SINAN database, we identified 46 456 cases of leprosy among 37 385 406 individuals who entered the 100 Million Brazilian Cohort between 2007 and 2014 (representing 16·4% of all 282 733 new cases of leprosy recorded in SINAN in that period; figure 2). After excluding 23 547 participants who did not meet the inclusion criteria, we also excluded those who met the exclusion criteria (n=11 453). The study sample included only the 11 456 patients with newly diagnosed leprosy (24·6% of the 46 456 linked cases) who were diagnosed with leprosy after entering the 100 Million Brazilian Cohort and met all other eligibility criteria.

8750 (76·4%) of 11 456 patients with leprosy began receiving PBF aid before their diagnosis and 2706 (23·6%) patients were not PBF beneficiaries by the time of leprosy diagnosis, treatment completion, or dropout. Relative to the 11 453 patients with newly diagnosed leprosy who were not included in the analysis because of being partial benefit receivers or having missing data or an income greater than BRL200 (figure 2), the included patients were younger, with a higher proportion of women and lower monthly familial and per-capita income (appendix pp 1, 2). PBF beneficiaries and non-beneficiaries differed by demographic and socioeconomic characteristics at entry to the 100 Million Brazilian Cohort (table 1).

**Table 1:** Characteristics of patients with newly diagnosed leprosy who entered the 100 Million Brazilian Cohort between 2007 and 2014, according to receipt of PBF aid

		**Non-beneficiaries (n=2706)**	**Beneficiaries (n=8750)**	p value*
**Individual variables**				
Age at registration, years		39·1 (26·7–53·6)	29·5 (20·3–40·7)	<0·0001
Age at diagnosis, years		41·4 (28·6–55·6)	32·9 (23·4–44·0)	<0·0001
Children		..	..	<0·0001
	Younger than 15 years	202/1726 (11·7%)	1524/1726 (88·3%)	..
	Aged 15 years and older	2504/9730 (25·7%)	7226/9730 (74·3%)	..
Sex		..	..	0·0026
	Male	1344 (49·7%)	4054 (46·3%)	..
	Female	1362 (50·3%)	4696 (53·7%)	..
Race or ethnicity		..	..	<0·0001
	White	528 (19·5%)	1356 (15·5%)	..
	Non-white (Asian, Indigenous, Black, and Mixed Black)	2133 (78·8%)	7272 (83·1%)	..
	Missing data	45 (1·7%)	122 (1·4%)	..
Education		..	..	<0·0001
	Illiterate	421 (15·6%)	1214 (13·9%)	..
	Primary school or less (≤5 years of education)	1018 (37·6%)	3273 (37·4%)	..
	Junior high school (≤9 years of education)	624 (23·1%)	2530 (28·9%)	..
	High school (≥10 years of education)	311 (11·5%)	821 (9·4%)	..
	Missing data	332 (12·3%)	912 (10·4%)	..
Work		..	..	<0·0001
	Employed	1289 (47·6%)	4802 (54·9%)	..
	Unemployed	1392 (51·4%)	3828 (43·7%)	..
	Missing	25 (0·9%)	120 (1·4%)	..
**Clinical variables**				
Operational disease classification		..	..	0·020
	Paucibacillary	1161 (42·9%)	3979 (45·5%)	..
	Multibacillary	1545 (57·1%)	4771 (54·5%)	..
Disabilities at diagnosis		..	..	0·059
	Grade 0	1754 (64·8%)	5904 (67·5%)	..
	Grade 1	566 (20·9%)	1644 (18·8%)	..
	Grade 2	147 (5·4%)	454 (5·2%)	..
	Not evaluated or missing	239 (8·8%)	748 (8·5%)	..
**Familial variables**				
Region of residence		..	..	<0·0001
	North	639 (23·6%)	2310 (26·4%)	..
	Northeast	1108 (40·9%)	3887 (44·4%)	..
	Southeast	362 (13·4%)	1165 (13·3%)	..
	South	86 (3·2%)	136 (1·6%)	..
	Midwest	511 (18·9%)	1252 (14·3%)	..
Area of residence		..	..	<0·0001
	Urban	2193 (81·0%)	6613 (75·6%)	..
	Rural	513 (19·0%)	2137 (24·4%)	..
Type of household		..	..	0·023
	Private	2663 (98·4%)	8545 (97·7%)	..
	Shared and informal housing	43 (1·6%)	205 (2·3%)	..
Construction material of household		..	..	<0·0001
	Bricks or cement	1954 (72·2%)	5687 (65·0%)	..
	Wood, other vegetal materials, or other	752 (27·8%)	3063 (35·0%)	..
Water supply		..	..	<0·0001
	Public network (tap water)	1929 (71·3%)	5520 (63·1%)	..
	Well, natural sources, or other	777 (28·7%)	3230 (36·9%)	..
Electricity		..	..	<0·0001
	Yes (with counter)	2347 (86·7%)	6899 (78·8%)	..
	Electricity without counter or no electricity	359 (13·3%)	1851 (21·2%)	..
Sewerage		..	..	<0·0001
	Public network or septic tank	1479 (54·7%)	4241 (48·5%)	..
	Homemade septic tank, ditch, or other	1227 (45·3%)	4509 (51·5%)	..
Waste		..	..	<0·0001
	Public collection system	2122 (78·4%)	6255 (71·5%)	..
	Burned, buried, outdoor disposal, or other	584 (21·6%)	2495 (28·5%)	..
Individuals per family		3 (2–4)	3 (2–4)	<0·0001
Residents per room		0·67 (0·50–1·00)	1·00 (0·67–1·33)	<0·0001
Monthly family income, BRL		200 (100–390)	150 (80–270)	<0·0001
Monthly per-capita income adjusted for PBF threshold, BRL†		80·0 (40·0–136·8)	43·3 (23·3–70·0)	<0·0001

Data are n (%) or median (IQR). Leprosy-related disabilities at diagnosis were classified as grade 0 if there was no presence of visible disabilities, grade 1 if there were signs of eye problems or anaesthesia in the hands and feet, and grade 2 if there was severe visual impairment or visible deformity or damage in the hands and feet. PBF=Programa Bolsa Família.

* p values calculated for a two-tailed *t* test for comparison of continuous variables and Pearson's χ^2^ for categorical variables.

† As the income threshold for eligibility increased by 1·167 in August, 2009, the monthly per-capita income was divided by 1·167 for families registering with CadÚnico after August, 2009, in this study.

5140 (44·9%) of 11 456 leprosy cases were paucibacillary leprosy and 6316 (55·1%) were multibacillary leprosy (table 2). Of 1726 children younger than 15 years, 1089 (63·1%) had paucibacillary leprosy and 637 (36·9%) had multibacillary leprosy (table 2). Treatment adherence in the cohort was high overall, and higher in paucibacillary cases than in multibacillary cases (table 2). Overall, 10 077 (88·0%) individuals were reported to have been cured of leprosy in SINAN. A higher proportion of patients with paucibacillary disease than with multibacillary disease were classified as cured (91·1% *vs* 85·4%). Rates of treatment adherence and cure were similar for children, for individuals of all ages overall, and for disease operational classification (table 2).

**Table 2:** Treatment duration, adherence, and cure in patients newly diagnosed with leprosy registered in the 100 Million Brazilian Cohort from 2007 to 2014, according to operational disease classification

		**Total**	**Paucibacillary**	**Multibacillary**
**Overall population**				
Patients, n		11 456	5140	6316
Months between diagnosis and end of treatment		10·1 (5·9–12·4)	6·1 (5·5–7·4)	11·8 (10·8–13·3)
Treatment adherence*				
	No	1948 (17·0%)	648 (12·6%)	1300 (20·6%)
	Yes	9508 (83·0%)	4492 (87·4%)	5016 (79·4%)
Cure				
	No (death, transfer of health unit, default)	1379 (12·0%)	459 (8·9%)	920 (14·6%)
	Yes	10 077 (88·0%)	4681 (91·1%)	5396 (85·4%)
**Children younger than 15 years**				
Patients, n		1726	1089	637
Months between diagnosis and end of treatment		7·0 (5·7–11·7)	6·1 (5·4–7·1)	12·1 (10·9–13·6)
Treatment adherence*				
	No	226 (13·1%)	125 (11·5%)	101 (15·9%)
	Yes	1500 (86·9%)	964 (88·5%)	536 (84·1%)
Cure				
	No (death, transfer of health unit, default)	169 (9·8%)	94 (8·6%)	75 (11·8%)
	Yes	1557 (90·2%)	995 (91·4%)	562 (88·2%)

Data are n, n (%), or median (IQR).

* Completing the minimum number of doses (at least six for paucibacillary cases and at least 12 for multibacillary cases).

After estimating the propensity score for receiving PBF on the basis of selected covariates for the overall population and for children younger than 15 years (appendix pp 4–7), we observed sufficient overlap in the propensity score distributions for PBF beneficiaries and non-beneficiaries (appendix p 12). Matching by propensity score resulted in 98·9% successfully matched pairs (8651 of 8750 beneficiaries) in the general population and 87·1% (1327 of 1524 beneficiaries) in children. Matching substantially increased the similarity between PBF and non-PBF recipients: the standardised mean difference between both groups was lower than 0·1 for most covariates (appendix pp 7, 8).

In the overall matched dataset including all ages, patients receiving PBF benefits before leprosy diagnosis had better treatment adherence than those who did not (OR 1·22, 95% CI 1·01–1·48). Similarly, PBF beneficiaries had higher odds of leprosy cure than non-beneficiaries (1·26, 1·01–1·58; table 3).

**Table 3:** Average treatment effect of PBF aid on leprosy treatment adherence and cure for the study cohort (Brazil, 2007–14)

		**Propensity score matched analysis**			**IPTW analysis**		
		Total	Paucibacillary	Multibacillary	Total	Paucibacillary	Multibacillary
**Overall population**							
Patients, n		17 302	7710	9336	11 456	5140	6316
Treatment adherence*							
	Non-beneficiaries	1·00	1·00	1·00	1·00	1·00	1·00
	Beneficiaries	1·22 (1·01–1·48)	1·37 (0·98–1·91)	1·37 (1·08–1·74)	1·31 (1·10–1·56)	1·29 (0·98–1·71)	1·38 (1·10–1·73)
Cure							
	Non-beneficiaries	1·00	1·00	1·00	1·00	1·00	1·00
	Beneficiaries	1·26 (1·01–1·58)	1·12 (0·75–1·67)	1·43 (1·09–1·90)	1·24 (1·00–1·52)	1·11 (0·79–1·55)	1·36 (1·05–1·77)
**Children younger than 15 years**							
Patients, n		2654	1466	722	1726	1089	637
Treatment adherence*							
	Non-beneficiaries	1·00	1·00	1·00	1·00	1·00	1·00
	Beneficiaries	1·55 (0·89–2·68)	1·91 (0·93–3·92)	1·47 (0·62–3·48)	1·71 (0·95–3·09)	1·77 (0·82–3·81)	1·41 (0·60–3·31)
Cure							
	Non-beneficiaries	1·00	1·00	1·00	1·00	1·00	1·00
	Beneficiaries	1·57 (0·83–2·97)	1·08 (0·41–2·82)	1·68 (0·67–4·24)	1·70 (0·84–3·43)	1·34 (0·48–3·72)	1·55 (0·61–3·99)

Data are n or OR (95% CI). The average treatment effect on the treated was estimated in the propensity score matched primary analysis and the IPTW sensitivity analysis. OR and 95% CI estimated using logistic regression with further adjustment for income. IPTW=inverse probability of treatment weighting. PBF=Programa Bolsa Família.

* Completing the minimum number of doses (at least six for paucibacillary cases and at least 12 for multibacillary cases).

In multibacillary cases, PBF exposure was associated with increased treatment adherence (ie, receiving ≥12 multidrug therapy doses) and cure (table 3). In paucibacillary cases, PBF exposure was not significantly associated with treatment adherence (ie, receiving at least six multidrug therapy doses) or cure. In the subgroup analysis of 1327 matched pairs of children younger than 15 years at diagnosis, there was no association between PBF exposure and leprosy treatment adherence or cure (table 3).

The sensitivity analysis with IPTW yielded similar results to the propensity-score matched analysis, with no indication of differences in the associations between PBF and adherence and cure in the overall population and in children (table 3). In the complete case analysis, we included only the 9960 individuals without missing data for any of the propensity score covariates. After matching with replacement (n=15 130), we detected no associations with treatment adherence (OR 1·17, 95% CI 0·95–1·43) or cure (1·20, 0·94–1·52), but analysis using IPTW yielded similar results to the primary analysis (appendix p 9).

We did further sensitivity analyses excluding 662 individuals (6% of the original cohort) who had not completed the minimum recommended number of doses but were considered cured by the clinician (appendix p 10). Using propensity-score matching, we found no evidence for an association between receiving PBF and treatment adherence in the overall population (OR 1·14, 95% CI 0·91–1·43). However, an association between between receipt of PBF and treatment adherence remained for multibacillary cases (1·48, 1·14–1·90). Similarly, the association between receiving PBF and leprosy cure was lost in the overall population (1·08, 0·87–1·35), but the association of PBF with cure remained evident for multibacillary cases (1·40, 1·09–1·79). Results were consistent between propensity-score matching and IPTW analyses (appendix p 10).

## Discussion

Using data on more than 11 000 patients with leprosy participating in the 100 Million Brazilian Cohort, we investigated the effect of a conditional cash transfer programme on leprosy treatment outcomes. Overall, we found evidence that PBF participation was associated with a 22% improvement in leprosy treatment adherence and 26% improvement in cure if enrolment in PBF occurred before diagnosis.

In patients with multibacillary disease who were receiving PBF benefits, we observed 43% higher cure rates than in patients with multibacillary disease who were non-beneficiaries. This finding is of potentially high public health importance as, relative to paucibacillary cases, multibacillary cases require longer treatment durations and, in our study, had lower cure rates overall (91·1% for paucibacillary *vs* 85·4% for multibacillary cases, table 2). Notably, the multibacillary cure rates were below the targets for cure (>90%) set by Brazilian states and municipalities.^28^ Moreover, multibacillary cases are associated with greater risks of transmission and progression to disabilities than are paucibacillary cases.^29^ Although relapses of leprosy disease are rare, they are most commonly reported in patients with multibacillary disease and have the potential to increase antimicrobial resistance.^30^ Hence, improvements to multibacillary treatment adherence and cure are of particular value for reducing the public health burden attributed to leprosy.

Our results add to the scant evidence base on the effects of social policies on leprosy, which currently consists of two ecological studies done in Brazil.^16^, ^17^ One study found the leprosy incidence in children younger than 15 years to be 15% lower in municipalities with high PBF coverage (≥50% coverage) than in municipalities with low coverage after adjusting for primary care coverage and socioeconomic characteristics.^17^ The other study reported leprosy incidence in the overall population to be up to 21% lower than in municipalities with low coverage when PBF coverage was maintained at high levels (>48%) for more than 4 years.^16^

Our findings align with results from previous studies investigating the effects of social protection programmes on treatment outcomes for other infectious diseases.^11^, ^12^, ^13^ A 2015 Liberian study of a community health worker programme providing transportation reimbursements, food support, and cash transfers found that it facilitated access to care and treatment adherence for patients with tuberculosis and HIV, although the study had insufficient power to analyse the effect of the programme on patients with leprosy.^31^ Furthermore, a systematic review and meta-analysis of nine social protection programmes, of which two were cash transfer programmes, showed a 37% reduction in risk of default on tuberculosis treatment and an 11% increase in tuberculosis cure rate in beneficiaries.^14^ Moreover, the PBF specifically has been associated with improved tuberculosis treatment outcomes in several studies.^11^, ^12^, ^13^ In two studies using CadÚnico and tuberculosis registry data and propensity score matching analyses, PBF receipt was associated with an increased tuberculosis cure rate.^11^, ^12^ Similarly, a multicentre prospective study done across seven Brazilian cities found that PBF beneficiaries had a higher tuberculosis cure rate and a lower risk of treatment dropout and death than propensity score-matched non-beneficiaries.^13^

It is well established that leprosy treatment outcomes are affected by factors related to socioeconomic status and health services (ie, quality and access). A 2013 systematic review identified illiteracy, low monthly per-capita income, low socioeconomic status, and poor knowledge of the disease as key factors related to leprosy treatment dropout.^7^ Additional factors include living in small households, migration, and working under precarious conditions.^3^ Given that more deprived individuals are more likely to receive benefits from the PBF, we would expect that those individuals, in the absence of PBF (as a counterfactual), would have had inferior rather than superior (as observed in our study) probabilities of adherance to multidrug therapy and of cure.

In Brazil, PBF has been shown to have a positive effect on various socioeconomic indicators through increased food security, educational attainment, and general improvement of the economic conditions of the household.^32^, ^33^, ^34^ Furthermore, although the PBF includes health-related and education-related conditions targeting children aged 0–7 years and pregnant and lactating women (ie, vaccinations, routine medical check-ups, and enrolment and attendance in school), the effect of increased use of health care has been suggested to extend beyond the individuals directly targeted by the programme.^35^ Although it is plausible that families who adhere to the PBF conditionalities are more likely than those who do not receive PBF aid to comply with a leprosy treatment programme, we would not expect this relationship to substantively confound the observed associations because families are eligible for the PBF benefit on the basis of their socioeconomic need and will only lose the PBF benefit at a minimum of 2 years of non-compliance with the conditions. Therefore, PBF receipt in the beneficiary group of our study does not correlate directly with individual families' compliant behaviours during the timescale of our analysis. Moreover, evidence from other social programmes, such as the initiatives in Mexico and Liberia, suggests that conditional cash transfers might have other ancillary benefits, such as reduced migration and improved access to health care, which could facilitate treatment adherence.^31^, ^36^ Furthermore, although the amount of cash transfered is small (BRL77–336 [$19–84] per family in 2014), preliminary evidence suggests that conditional cash transfer programmes can alleviate the financial burden of families affected by leprosy, similar to what has been observed in those affected by tuberculosis.^6^, ^37^

In 2016, a randomised trial in tuberculosis-affected households in Peru showed that those receiving cash transfers were 12% less likely to incur out-of-pocket tuberculosis-related expenses that led to impoverishment than those who did not receive transfers.^37^ Another mechanism by which the PBF might directly improve treatment adherence is through the conditionality related to child attendance at routine health check-ups. In our analysis, the point estimates for the association between PBF receipt and treatment adherence and cure were higher in children younger than 15 years than in the overall population (table 3). Nevertheless, these associations were not significant, which might be explained by the small sample size and low frequency of study outcomes. Future research is needed to investigate why the point estimates for children were higher than those for the overall population. Overall, the association of PBF with leprosy treatment adherence and cure is compatible with the theory that social development was one of the main mechanisms responsible for leprosy elimination in the first half of the 20th century in high-income countries.^38^

Our study was possible because of the use of nationwide linked datasets and the relatively high endemicity of leprosy in Brazil, enabling us to study more than 11 000 patients with leprosy. Because of the large sample size, we were able to stratify by type of leprosy (paucibacillary and multibacillary), which affects the treatment durations. The large sample size also allowed us to investigate the effect of PBF on leprosy treatment outcomes in children, a group of high priority in leprosy control strategies. As the 100 Million Brazilian Cohort contained information about the poorest families in the Brazilian population, our study was a unique opportunity to study a social protection policy targeting this group and verify its effect on a disease that primarily affects the poorest and most vulnerable population of the country. 5, 8

There are, however, limitations to our study. First, as expected using secondary data, the proportion of missing data for variables, such as education and work status, was notable (>10%). To account for missing data as a potential confounder, we included a missing category for individual sociodemographic variables. Nevertheless, complete case analysis showed weakened associations between PBF receipt and treatment adherence or cure, but with similar point estimates for patients with multibacillary disease, suggesting that the exclusions for missing data did not substantially alter our findings for multibacillary cases. Although we were able to control for the key sociodemographic confounders, residual confounding is possible because data on other factors related to treatment adherence, including behavioural characteristics (eg, drug use and alcohol misuse) and access to and quality of health services (eg, distance to clinic or skill and preparedness of health professionals), were not available in the routinely collected datasets linked in this cohort.^7^, ^16^

In conclusion, our findings suggest that tackling adverse socioeconomic factors in patients with leprosy should be central to strategies for improving cure rates and ultimately eliminating the disease. We provide new evidence that programmes such as the PBF, which directly support patients of low socioeconomic status, have the potential to improve treatment adherence and cure in patients with leprosy. In the context of political instability and implementation of several austerity measures in Brazil, including the exclusion of more than 1 million families from the PBF in 2017, budget restrictions to the Brasil sem Miséria social programme implemented in 2011, which complements the benefits from PBF, and the Constitutional Amendment 95 (EC95) in 2016, which limits any real growth in federal expenditures on public health care for the next 20 years, social security in Brazil is under threat,^39^ which might jeopardise the indisputable health gains achieved from PBF's implementation to date.^40^ Therefore, on the basis of the evidence provided by our study, we recommend that poverty-alleviating programmes, such as the PBF, should be viewed not only as essential tools to improve the wellbeing of poor families in Brazil, but also as essential components of WHO's Global Leprosy Strategy 2016–2020, which advocates for access to social and financial support services and strengthened relationships between patients and health services.^1^ Additionally, these programmes should be considered as important contributors to the achievement of the Sustainable Development Goals for neglected tropical diseases (goal 3, target 3.3).

**Contributors**

JMP, MYI, JSN, MLFP, RLF, LCR, GOP, and MLB developed the study concept. MYI, JSN, GOP, and MLB collected the data. JMP, EW, RLF, AR, and EBB designed the study and investigation. JMP, EW, AR, and EBB analysed the data and created the figures. JMP, JSN, AR, and EBB wrote the first draft of the manuscript, and all authors reviewed and edited the manuscript. LS, LCR, EBB, GOP, and MLB supervised the study process. MYI, RLF, JSN, AS, LS, LCR, GOP, and MLB acquired funding.

**Declaration of interests**

JMP reports personal fees from CONFAP/ESRC/MRC/BBSRC/FAPDF during the conduct of the study. JSN reports grants from Coordenação de Aperfeiçoamento de Pessoal de Nível Superior–Brazil (CAPES) during the conduct of the study. LS reports grants from the Wellcome Trust during the conduct of the study; grants from the Medical Research Council (MRC), National Institute for Health Research, GlaxoSmithKline, British Heart Foundation (BHS), and Diabetes UK, outside the submitted work; and is a trustee of the BHS. AR, EBB, and LCR report grants from the MRC during the conduct of the study. EW, RLF, GOP, MYI, and MLB declare no competing interests.

## References

[1] WHO (2016). Global Leprosy Strategy 2016–2020: accelerating towards a leprosy-free world: operational manual.

[2] Mitra AK, Mawson AR (2017). Neglected tropical diseases: epidemiology and global burden. Trop Med Inf Dis.

[3] Heukelbach J, André Chichava O, de Oliveira AR (2011). Interruption and defaulting of multidrug therapy against leprosy: population-based study in Brazil's savannah region. PLoS Negl Trop Dis.

[4] Pescarini JM, Strina A, Nery JS (2018). Socioeconomic risk markers of leprosy in high-burden countries: a systematic review and meta-analysis. PLoS Negl Trop Dis.

[5] Nery JS, Ramond A, Pescarini JM (2019). Socioeconomic determinants of leprosy new case detection in the 100 Million Brazilian Cohort: a population-based linkage study. Lancet Glob Health.

[6] Xiong M, Li M, Zheng D (2017). Evaluation of the economic burden of leprosy among migrant and resident patients in Guangdong province, China. BMC Inf Dis.

[7] Girão RJS, Soares NLR, Pinheiro JV (2013). Leprosy treatment dropout: a systematic review. Int Arch Med.

[8] Soares FV (2011). Brazil's Bolsa Família: a review. Econ Polit Weekly.

[9] Gertler P (2000). Final report: the impact of PROGRESA on health.

[10] Shei A, Costa F, Reis MG, Ko AI (2014). The impact of Brazil's Bolsa Família conditional cash transfer program on children's health care utilization and health outcomes. BMC Int Health Hum Rights.

[11] Torrens AW, Rasella D, Boccia D (2016). Effectiveness of a conditional cash transfer programme on TB cure rate: a retrospective cohort study in Brazil. Trans R Soc Trop Med Hyg.

[12] Carter DJ, Daniel R, Torrens AW (2019). The impact of a cash transfer programme on tuberculosis treatment success rate: a quasi-experimental study in Brazil. BMJ Glob Health.

[13] Oliosi JGN, Reis-Santos B, Locatelli RL (2019). Effect of the Bolsa Familia Programme on the outcome of tuberculosis treatment: a prospective cohort study. Lancet Glob Health.

[14] Andrade KVF, Nery JS, Souza RA, Pereira SM (2018). Effects of social protection on tuberculosis treatment outcomes in low or middle-income and in high-burden countries: systematic review and meta-analysis. Cad Saude Publica.

[15] Wingfield T, Tovar MA, Huff D (2017). A randomized controlled study of socioeconomic support to enhance tuberculosis prevention and treatment, Peru. Bull World Health Org.

[16] Nery JS, Pereira SM, Rasella D (2014). Effect of the Brazilian conditional cash transfer and primary health care programs on the new case detection rate of leprosy. PLoS Negl Trop Dis.

[17] Andrade KVF, Nery JS, Penna ML, Penna GO, Pereira SM (2018). Effect of Brazil's conditional cash transfer programme on the new case detection rate of leprosy in children under 15 years old. Leprosy Rev.

[18] Soares S, De Souza PHGF, Osório RG, Silveira FG (2003). Os impactos do benefício do Programa Bolsa Família sobre a desigualdade e a pobreza. Bolsa Família.

[19] Pescarini JM, Alves A, Strina A, Cidacs (2019). Dataset—leprosy incidence and treatment outcomes in the 100 Million Brazilian Cohort. https://hdl.handle.net/20.500.12196/FK2/FNMRCA.

[20] CIDACS (2019). Coorte de 100 Milhões de Brasileiros. https://cidacs.bahia.fiocruz.br/en/platform/cohort-of-100-million-brazilians/.

[21] Campello T, Neri MC (2013). Programa Bolsa Família: uma década de inclusão e cidadania.

[22] Ali MS, Ichihara MY, Lopes LC (2019). Administrative Data linkage in Brazil: potentials for health technology assessment. Front Pharmacol.

[23] Pita R, Pinto C, Sena S (2018). On the accuracy and scalability of probabilistic data linkage over the Brazilian 114 Million Cohort. IEEE J Biomed Health Inform.

[24] Brasil, Ministério da Saúde, Secretaria de Vigilância em Saúde (2016). Diretrizes para vigilância, atenção e eliminação da Hanseníase como problema de saúde pública: manual técnico-operacional.

[25] Williamson E, Morley R, Lucas A, Carpenter J (2012). Propensity scores: from naive enthusiasm to intuitive understanding. Stat Methods Med Res.

[26] Hill J, Reiter JP (2006). Interval estimation for treatment effects using propensity score matching. Stat Med.

[27] Vieira MCA, Nery JS, Paixão ES, Freitas de Andrade KV, Oliveira Penna G, Teixeira MG (2018). Leprosy in children under 15 years of age in Brazil: a systematic review of the literature. PLoS Negl Trop Dis.

[28] Secretaria de Vigilância em Saúde do Distrito Federal (2017). Pactuação interfederativa 2017–2021: caderno de diretrizes, objetivos, metas e indicadores.

[29] Sales AM, Campos DP, Hacker MA (2013). Progression of leprosy disability after discharge: is multidrug therapy enough?. Trop Med Int Health.

[30] Cambau E, Saunderson P, Matsuoka M (2018). Antimicrobial resistance in leprosy: results of the first prospective open survey conducted by a WHO surveillance network for the period 2009–15. Clin Microb Infect.

[31] Rogers JH, Jabateh L, Beste J (2018). Impact of community-based adherence support on treatment outcomes for tuberculosis, leprosy and HIV/AIDS-infected individuals in post-Ebola Liberia. Glob Health Act.

[32] Martins APB, Monteiro CA (2016). Impact of the Bolsa Família program on food availability of low-income Brazilian families: a quasi experimental study. BMC Public Health.

[33] Simões AA, Sabates R (2014). The contribution of Bolsa Família to the educational achievement of economically disadvantaged children in Brazil. Int J Educ Dev.

[34] Santos TMd, Silva SSdC, Koller SH (2017). Evaluation of Amazon riverine beneficiaries about Bolsa Família Program. Psic: Teor e Pesq.

[35] Lagarde M, Haines A, Palmer N (2009). The impact of conditional cash transfers on health outcomes and use of health services in low and middle income countries. Cochrane Database Syst Rev.

[36] Stecklov G, Winters P, Stampini M, Davis B (2005). Do conditional cash transfers influence migration? A study using experimental data from the Mexican PROGRESA program. Demography.

[37] Wingfield T, Tovar MA, Huff D (2016). The economic effects of supporting tuberculosis-affected households in Peru. Eur Resp J.

[38] Lie H (1929). Why is leprosy decreasing in Norway?. Trans Royal Soc Trop Med Hyg.

[39] Paes-Sousa R, Rasella D, Carepa-Sousa J (2018). Economic policy and public health: fiscal balance and population wellbeing. Saúde em Debate.

[40] Rasella D, Basu S, Hone T, Paes-Sousa R, Ocké-Reis CO, Millett C (2018). Child morbidity and mortality associated with alternative policy responses to the economic crisis in Brazil: a nationwide microsimulation study. PLos Med.

