# Supplementary material for: Effect of a conditional cash transfer programme on leprosy treatment adherence and cure in patients from the nationwide 100 Million Brazilian Cohort: a quasi-experimental study
Source: Lancet Infect Dis. 2020 May;20(5):618–27. doi: 10.1016/S1473-3099(19)30624-3 (PMC7191267; doi:10.1016/S1473-3099(19)30624-3)
Supplement: Supplementary appendix [file mmc1.pdf]

THE LANCET Infectious Diseases

Supplementary webappendix

This webappendix formed part of the original submission and has been peer reviewed. We post it as supplied by the authors.

Supplement to: Pescarini JM, Williamson E, Nery JS, et al. Effect of a conditional cash transfer programme on leprosy treatment adherence and cure in patients from the nationwide 100 Million Brazilian Cohort: a quasi-experimental study. *Lancet Infect Dis* 2020; published online Feb 14. https://doi.org/10.1016/S1473-3099(19)30624-3.

Supplementary material

Table S1 Profile of new applicant families in Cadastro Único from 2007 to 2014 that had a new leprosy case in the same period prior and after exclusion of partial BFP receivers, individuals with missing data, and those with income >200 (N=22 909).

Social and demographical variables	N (%) or Median (IQR)		
	Overall (N=22 909)	Study sample (N=11 546)	Excluded sample (N=11 453)
Individual variables			
**Age at registration**	35·5 (25·0-53·2)	31·0 (22·1-44·4)	43·7 (28·3-61·5)
**Age at diagnosis**	38·5 (27·7-55·8)	34·0 (24·8-47·4)	46·3 (30·8-64·0)
**Sex**			
Males	11561 (50·5)	5398 (47·1)	6163 (53·8)
Females	11348 (49·5)	6058 (52·9)	5290 (46·2)
**Ethnicity**			
White	4282 (18·7)	1884 (16·4)	2398 (20·9)
Non-white (Asian, Indigenous, Blacks and Mixed Black)	18181 (79·4)	9405 (82·1)	8776 (76·6)
Missing	446 (1·9)	167 (1·5)	279 (2·4)
**Education**			
Illiterate	3844 (16·8)	1635 (14·3)	2209 (19·3)
Primary school or less (≤5 years of education)	8533 (37·2)	4291 (37·5)	4242 (37·0)
Junior high school (≤9 years of education)	5669 (24·7)	3154 (27·5)	2515 (22·0)
High school (≥10 years of education)	2322 (10·1)	1132 (9·9)	1190 (10·4)
Missing	2541 (11·1)	1244 (10·9)	1297 (11·3)
**Work**			
Employed	10063 (43·9)	6091 (53·2)	3972 (34·7)
Unemployed	10473 (45·7)	5220 (45·6)	5253 (45·9)
Missing	2373 (10·4)	145 (1·3)	2228 (19·5)
Clinical variables			
**Operational classification**			
Paucibacillary	9162 (40·0)	5140 (44·9)	4022 (35·1)
Multibacillary	13745 (60·0)	6316 (55·1)	7429 (64·9)
Missing	2 (0·0)	0 (0·0)	2 (0·0)
**Disabilities at diagnosis**			
Grade 0	14441 (63·0)	7658 (66·8)	6783 (59·2)
Grade 1	4852 (21·2)	2210 (19·3)	2642 (23·1)
Grade 2	1387 (6·1)	601 (5·2)	786 (6·9)
Not evaluated or missing	2229 (9·7)	987 (8·6)	1242 (10·8)
**Treatment adherence**[3]			
No	3871 (16·9)	1948 (17·0)	1923 (16·8)
Yes	17446 (76·2)	9508 (83·0)	7938 (69·3)
Missing	1592 (6·9)	0 (0·0)	1592 (13·9)
**Cure**			
No	2828 (12·3)	1379 (12·0)	1449 (12·7)

Yes	19003 (82·9)	10077 (88·0)	8926 (77·9)
Missing	1078 (4·7)	0 (0·0)	1078 (9·4)
Familial variables			
**Bolsa familia receipt**			
No	9369 (40·9)	2706 (23·6)	6663 (58·2)
Yes	13540 (59·1)	8750 (76·4)	4790 (41·8)
**Region of residence**			
North	5267 (23·0)	2949 (25·7)	2318 (20·2)
Northeast	9514 (41·5)	4995 (43·6)	4519 (39·5)
Southeast	3187 (13·9)	1527 (13·3)	1660 (14·5)
South	634 (2·8)	222 (1·9)	412 (3·6)
Midwest	4307 (18·8)	1763 (15·4)	2544 (22·2)
**Area of residence**			
Urban	18179 (79·4)	8806 (76·9)	9373 (81·8)
Rural	4709 (20·6)	2650 (23·1)	2059 (18·0)
Missing	21 (0·1)	0 (0·0)	21 (0·2)
**Type of the household**			
Private	20113 (87·8)	11208 (97·8)	8905 (77·8)
Shared and informal housing	666 (2·9)	248 (2·2)	418 (3·6)
Missing	2130 (9·3)	0 (0·0)	2130 (18·6)
**Construction material**			
Bricks/cement	15721 (68·6)	7641 (66·7)	8080 (70·5)
Wood, other vegetal materials, or other	6875 (30·0)	3815 (33·3)	3060 (26·7)
Missing	313 (1·4)	0 (0·0)	313 (2·7)
**Water supply (tap water)**			
Public network (tap water)	15446 (67·4)	7449 (65·0)	7997 (69·8)
Well, natural sources, or other	7150 (31·2)	4007 (35·0)	3143 (27·4)
Missing	313 (1·4)	0 (0·0)	313 (2·7)
**Electricity**			
Yes with counter	18957 (82·7)	9246 (80·7)	9711 (84·8)
Electricity without counter or no electricity	3639 (15·9)	2210 (19·3)	1429 (12·5)
**Missing**	313 (1·4)	0 (0·0)	313 (2·7)
**Sewage**			
Public network or septic tank	11801 (51·5)	5720 (49·9)	6081 (53·1)
Homemade septic tank, ditch, or other	10416 (45·5)	5736 (50·1)	4680 (40·9)
Missing	692 (3·0)	0 (0·0)	692 (6·0)
**Waste**			
Public collection system	17083 (74·6)	8377 (73·1)	8706 (76·0)
Burned, buried, outdoor disposal, or other	5513 (24·1)	3079 (26·9)	2434 (21·3)
Missing	313 (1·4)	0 (0·0)	313 (2·7)
**Individuals per family**	3 (2-4)	3 (2-4)	2 (1-3)
**Residents per room**	0·8 (0·4-1·0)	1·0 (0·6-1·3)	0·5 (0·3-1·0)
**Monthly family income (BRL)**	200 (100-465)	150 (80-300)	380 (120-622)
**Monthly per capita income (BRL) adjusted for BFP threshold**[1]	64·3 (31·0-171·4)	50 (25-85·7)	128·5 (42·8-380)

[1] As the income threshold for BFP eligibility increased by 1·167 in August 2009, the monthly per capita income was divided by 1·167 for families registering with CadUnico after August 2009 in this study.

Table S2 Logistic regression to estimate the probability of receiving BFP benefit according to covariates (N= 11 456).

**Social and demographical variables**	**Overall**	**PB**	**MB**
	OR (95% CI)	OR (95% CI)	OR (95% CI)
**Intercept**	3·92 (2·83-5·42)	3·98 (2·44-6·51)	4·08 (2·63-6·33)
Individual variables			
**Age at registration**	0·97 (0·97-0·98)	0·97 (0·97-0·98)	0·97 (0·97-0·98)
**Sex**			
Males	1·00	1·00	1·00
Females	1·09 (0·99-1·21)	1·17 (1-1·37)	1·1 (0·96-1·25)
**Ethnicity**			
White	1·00	1·00	1·00
Non-white (Asian, Indigenous, Blacks and Mixed Black)	1 (0·88-1·14)	1·11 (0·91-1·35)	0·93 (0·78-1·12)
Missing	1·71 (1·12-2·67)	2·50 (1·24-5·05)	1·32 (0·76-2·31)
**Education**			
Illiterate	1·00	1·00	1·00
Primary school or less (≤5 years of education)	1·09 (0·94-1·27)	0·93 (0·72-1·21)	1·19 (0·99-1·43)
Junior high school (≤9 years of education)	1·13 (0·96-1·33)	0·94 (0·71-1·23)	1·27 (1·03-1·58)
High school (≥10 years of education)	0·88 (0·71-1·07)	0·71 (0·52-0·97)	1·01 (0·76-1·34)
Missing	0·75 (0·62-0·91)	0·65 (0·48-0·88)	0·81 (0·63-1·06)
**Work**			
Employed	1·00	1·00	1·00
Unemployed	0·8 (0·72-0·89)	0·82 (0·69-0·96)	0·79 (0·68-0·92)
Missing	1·6 (0·98-2·69)	1·14 (0·51-2·56)	1·93 (1·01-3·69)
Familial variables			
**Region of residence**			
North	1·00	1·00	1·00
Northeast	1·28 (1·11-1·47)	1·27 (1·03-1·56)	1·29 (1·06-1·55)
Southeast	1·67 (1·38-2·01)	1·60 (1·22-2·11)	1·75 (1·36-2·25)
South	1·14 (0·82-1·61)	1·59 (0·77-3·29)	1·00 (0·68-1·49)
Midwest	1·31 (1·11-1·54)	1·33 (1·02-1·72)	1·27 (1·03-1·58)
**Area of residence**			
Urban	1·00	1·00	1·00
Rural	0·99 (0·84-1·17)	0·98 (0·76-1·26)	1·02 (0·82-1·27)
**Type of the household**			
Private	1·00	1·00	1·00
Shared and informal housing	1·07 (0·75-1·55)	0·99 (0·59-1·66)	1·16 (0·7-1·92)
**Construction material**			
Bricks/cement	1·00	1·00	1·00
Wood, other vegetal materials, or other	0·99 (0·87-1·13)	0·93 (0·76-1·13)	1·04 (0·88-1·23)
**Water supply (tap water)**			
Public network (tap water)	1·00	1·00	1·00
Well, natural sources, or other	1·16 (1·02-1·32)	1·06 (0·87-1·29)	1·23 (1·04-1·47)
**Electricity**			

Yes with counter	1·00	1·00	1·00
Electricity without counter or no electricity	1·08 (0·93-1·25)	1·1 (0·88-1·38)	1·07 (0·87-1·31)
**Sewage**			
Public network or septic tank	1·00	1·00	1·00
Homemade septic tank, ditch, or other	1·07 (0·96-1·2)	1·11 (0·94-1·31)	1·05 (0·91-1·22)
**Waste**			
Public collection system	1·00	1·00	1·00
Burned, buried, outdoor disposal, or other	0·9 (0·76-1·06)	0·88 (0·68-1·13)	0·91 (0·73-1·13)
**Residents per room (household density)**	1·53 (1·41-1·67)	1·55 (0·1-0)	1·51 (0·09-0)
**Per capita income (BRL) adjusted for BFP threshold**[1]			
0	1·00	1·00	1·00
0 -| 50	1·48 (1·25-1·77)	1·62 (1·25-2·11)	1·39 (1·1-1·75)
50 -| 100	0·81 (0·68-0·95)	0·79 (0·61-1·02)	0·83 (0·66-1·04)
100 -| 150	0·42 (0·34-0·5)	0·4 (0·3-0·54)	0·43 (0·33-0·55)
150 -| 200	0·14 (0·11-0·17)	0·14 (0·1-0·19)	0·14 (0·1-0·19)
**Year of registry**			
2007	1·00	1·00	1·00
2008	1·42 (1·25-1·61)	1·31 (1·08-1·58)	1·54 (1·3-1·84)
2009	2·62 (2·24-3·07)	2·43 (1·92-3·07)	2·82 (2·28-3·49)
2010	2·01 (1·64-2·49)	2·13 (1·53-2·95)	1·97 (1·5-2·59)
2011	1·13 (0·89-1·45)	1·14 (0·77-1·68)	1·15 (0·84-1·59)
2012	0·74 (0·61-0·91)	0·57 (0·41-0·77)	0·91 (0·69-1·2)
2013	0·56 (0·4-0·77)	0·68 (0·41-1·12)	0·49 (0·32-0·74)
2014	0·35 (0·2-0·6)	0·16 (0·06-0·44)	0·53 (0·27-1·04)

MB: multibacillary, PB: paucibacillary, OR: odds ratio

[1] As the income threshold for BFP eligibility increased by 1·167 in August 2009, the monthly per capita income was divided by 1·167 for families registering with CadUnico after August 2009 in this study.

Table S3 Logistic regression to estimate the probability of receiving BFP benefit according to covariates for children under 15 (N= 1726).

Social and demographical variables	Overall	PB	MB
	OR (95% CI)	OR (95% CI)	OR (95% CI)
**Intercept**	1·41 (0·41-5·06)	1·07 (0·22-5·31)	3·11 (0·27-35·76)
Individual variables			
**Age at diagnosis**			
0-4	1·00	1·00	1·00
5-9	1·76 (0·85-3·42)	2·26 (1·03-4·97)	0·85 (0·16-4·44)
10-14	1·38 (0·68-2·62)	1·84 (0·86-3·96)	0·73 (0·14-3·7)
**Sex**			
Males	1·00	1·00	1·00
Females	1·05 (0·76-1·46)	1·19 (0·78-1·82)	0·80 (0·45-1·4)
**Ethnicity**			
White	1·00	1·00	1·00
Non-white	0·76 (0·45-1·26)	0·56 (0·28-1·12)	1·19 (0·5-2·84)
Missing	0·10 (0·02-0·72)	0·08 (0·01-0·83)	0·16 (0-5·99)
**Education (head)**			
Illiterate	1·00	1·00	1·00
Primary school or less (≤5 years of education)	1·56 (0·92-2·61)	1·77 (0·87-3·6)	1·08 (0·48-2·43)
Junior high school (≤9 years of education)	1·53 (0·89-2·6)	1·67 (0·81-3·41)	1·32 (0·55-3·18)
High school (≥10 years of education)	1·30 (0·67-2·51)	1·33 (0·57-3·08)	1·37 (0·41-4·64)
Missing	0·90 (0·48-1·68)	1 (0·44-2·28)	0·64 (0·22-1·9)
**Work (head)**			
Employed	1·00	1·00	1·00
Unemployed	0·95 (0·66-1·4)	0·91 (0·56-1·47)	0·95 (0·49-1·85)
Missing	1·71 (0·34-11·38)	3·05 (0·27-34·78)	0·30 (0·01-6·67)
Familial variables			
**Region of residence**			
North	1·00	1·00	1·00
Northeast	2·48 (1·61-3·83)	2·51 (1·42-4·41)	2·77 (1·35-5·71)
Southeast	3·17 (1·7-6·15)	3·29 (1·5-7·22)	2·30 (0·7-7·6)
South	1·02 (0·21-6·42)	2·22 (0·18-27·16)	0·14 (0·01-2·69)
Midwest	2·19 (1·25-3·97)	1·8 (0·86-3·77)	3·49 (1·21-10·03)
**Area of residence**			
Urban	1·00	1·00	1·00
Rural	0·98 (0·57-1·71)	1·44 (0·69-2·98)	0·43 (0·18-1·04)
**Type of the household**			
Private	1·00	1·00	1·00
Shared and informal housing	1·25 (0·44-4·53)	0·78 (0·23-2·68)	1 (0-0)
**Construction material**			
Bricks/cement	1·00	1·00	1·00
Wood, other vegetal materials, or other	1·27 (0·83-1·94)	1·21 (0·7-2·09)	1·38 (0·65-2·94)
**Water supply (tap water)**			

Public network (tap water)	1·00	1·00	1·00
Well, natural sources, or other	0·90 (0·60-1·36)	0·92 (0·54-1·59)	0·74 (0·38-1·44)
**Electricity**			
Yes with counter	1·00	1·00	1·00
Electricity without counter or no electricity	1·64 (1·01-2·74)	1·39 (0·74-2·6)	2·68 (1·11-6·46)
**Sewage**			
Public network or septic tank	1·00	1·00	1·00
Homemade septic tank, ditch, or other	1·25 (0·86-1·81)	1·26 (0·79-2·03)	1·50 (0·78-2·88)
**Waste**			
Public collection system	1·00	1·00	1·00
Burned, buried, outdoor disposal, or other	0·66 (0·38-1·15)	0·58 (0·29-1·18)	0·86 (0·36-2·1)
**Residents per room (household density)**	1·39 (1·13-1·75)	1·59 (0·26-0)	1·23 (0·18-0)
**Per capita income (BRL) adjusted for BFP threshold**[1]			
0	1·00	1·00	1·00
0 -| 50	1·23 (0·64-2·3)	1·6 (0·72-3·55)	0·76 (0·24-2·41)
50 -| 100	0·62 (0·32-1·16)	0·66 (0·3-1·44)	0·53 (0·16-1·8)
100 -| 150	0·32 (0·15-0·69)	0·37 (0·14-0·96)	0·23 (0·06-0·95)
150 -| 200	0·02 (0·01-0·06)	0·03 (0·01-0·1)	0 (0-0·04)
**Year of registry**			
2007	1·00	1·00	1·00
2008	1·93 (1·27-2·99)	1·67 (0·98-2·85)	2·93 (1·32-6·51)
2009	4·86 (2·61-9·82)	5·14 (2·16-12·24)	5·43 (1·76-16·77)
2010	4·82 (2·13-12·69)	4·09 (1·43-11·66)	8·64 (1·26-59·20)
2011	1·02 (0·42-2·77)	0·80 (0·25-2·57)	2·30 (0·33-16·11)
2012	1·73 (0·77-4·25)	1·96 (0·65-5·84)	1·06 (0·26-4·33)
2013	1·69 (0·52-6·34)	1·63 (0·36-7·42)	1·24 (0·08-18·11)
2014	0·38 (0·07-2·47)	0·08 (0·01-0·91)	6·55 (0·15-289·33)

MB: multibacillary, PB: paucibacillary, OR: odds ratio

[1] As the income threshold for BFP eligibility increased by 1·167 in August 2009, the monthly per capita income was divided by 1·167 for families registering with CadUnico after August 2009 in this study.

Table S4 Standardized mean differences (SMD) before and after matching for the matching covariates and those used in further adjustment in the overall population.

	Before matching (N= 11 456)			After matching (overall) (N= 17 302)		
	Non-BFP	BFP		Non-BFP	BFP	
	N or mean (% or sd)	N or mean (% or sd)	SMD	N or mean (% or sd)	N or mean (% or sd)	SMD
**Age at registration**	39·7 (17·2)	30 (15·9)	0·586	30·1 (15·4)	30·2 (15·9)	-0·006
**Sex**						
Males	1344 (49·7)	4054 (46·3)	0·067	4208 (48·6)	4009 (46·3)	0·046
Females	1362 (50·3)	4696 (53·7)		4443 (51·4)	4642 (53·7)	
**Ethnicity**						
White	528 (19·5)	1356 (15·5)	0·109	1310 (15·1)	1347 (15·6)	0·028
Non-white	2133 (78·8)	7272 (83·1)		7245 (83·7)	7184 (83)	
Missing	45 (1·7)	122 (1·4)		96 (1·1)	120 (1·4)	
**Education**						
Illiterate	421 (15·6)	1214 (13·9)	0·150	1407 (16·3)	1201 (13·9)	0·074
Primary school or less (≤5 years of education)	1018 (37·6)	3273 (37·4)		3021 (34·9)	3216 (37·2)	
Junior high school (≤9 years of education)	624 (23·1)	2530 (28·9)		2519 (29·1)	2505 (29)	
High school (≥10 years of education)	311 (11·5)	821 (9·4)		782 (9)	820 (9·5)	
Missing	332 (12·3)	912 (10·4)		922 (10·7)	909 (10·5)	
**Work**						
Employed	1289 (47·6)	4802 (54·9)	0·157	4757 (55)	4734 (54·7)	0·038
Unemployed	1392 (51·4)	1380 (15·8)		3736 (43·2)	3799 (43·9)	
Missing	25 (0·9)	120 (1·4)		158 (1·8)	118 (1·4)	
**Region of residence**						
North	639 (23·6)	2310 (26·4)	0·173	2837 (32·8)	2256 (26·1)	0·150
Northeast	1108 (40·9)	3887 (44·4)		3584 (41·4)	3861 (44·6)	
Southeast	362 (13·4)	1165 (13·3)		1021 (11·8)	1153 (13·3)	
South	86 (3·2)	136 (1·6)		129 (1·5)	135 (1·6)	
Midwest	511 (18·9)	1252 (14·3)		1080 (12·5)	1246 (14·4)	
**Area of residence**						
Urban	2193 (81)	6613 (75·6)	0·133	6339 (73·3)	6543 (75·6)	0·054
Rural	513 (19)	2137 (24·4)		2312 (26·7)	2108 (24·4)	
**Type of the household**						
Private	2663 (98·4)	8545 (97·7)	0·054	8508 (98·3)	8449 (97·7)	0·049
Shared and informal housing	43 (1·6)	205 (2·3)		143 (1·7)	202 (2·3)	
**Construction material**						
Bricks/cement	1954 (72·2)	5687 (65)	0·156	5361 (62)	5642 (65·2)	0·068
Wood, other vegetal materials, or other	752 (27·8)	3063 (35)		3290 (38)	3009 (34·8)	
**Water supply (tap water)**						
Public network (tap water)	1929 (71·3)	5520 (63·1)	0·175	5299 (61·3)	5483 (63·4)	0·044
Well, natural sources, or other	777 (28·7)	3230 (36·9)		3352 (38·7)	3168 (36·6)	
**Electricity**						

Yes with counter	2347 (86·7)	6899 (78·8)	0·210	6695 (77·4)	6853 (79·2)	0·044
Electricity without counter or no electricity	359 (13·3)	1851 (21·2)		1956 (22·6)	1798 (20·8)	
**Sewage**						
Public network or septic tank	1479 (54·7)	4241 (48·5)	0·124	4146 (47·9)	4212 (48·7)	0·015
Homemade septic tank, ditch, or other	1227 (45·3)	4509 (51·5)		4505 (52·1)	4439 (51·3)	
**Waste**						
Public collection system	2122 (78·4)	6255 (71·5)	0·161	6068 (70·1)	6195 (71·6)	0·032
Burned, buried, outdoor disposal, or other	584 (21·6)	2495 (28·5)		2583 (29·9)	2456 (28·4)	
**Residents per room**	0·8 (0·65)	1·2 (0·94)	-0·443	1·2 (1·1)	1·1 (0·83)	0·107
Per capita income (BRL) adjusted for BFP threshold[1]						
0	342 (12·6)	1076 (12·3)	0·761	1144 (13·2)	1075 (12·4)	0·052
0 -| 50	622 (23·)	4264 (48·7)		4208 (48·6)	4174 (48·2)	
50 -| 100	743 (27·5)	2450 (28·0)		2347 (27·1)	2443 (28·2)	
100 -| 150	485 (17·9)	741 (8·5)		683 (7·9)	741 (8·6)	
150 -| 200	514 (19·0)	219 (2·5)				
Per capita income (BRL) adjusted for BFP threshold[1] **(cont)**	86·8 (60·6)	51·1 (40·3)	0·693	53·8 (42·7)	51·4 (40·4)	0·058
**Year of registry**						
2007	1196 (44·2)	3842 (43·9)	0·428	3865 (44·7)	3806 (44·0)	0·141
2008	517 (19·1)	1963 (22·4)		2351 (27·2)	1940 (22·4)	
2009	278 (10·3)	1587 (18·1)		1251 (14·5)	1556 (18·0)	
2010	158 (5·8)	670 (7·7)		549 (6·3)	663 (7·7)	
2011	146 (5·4)	251 (2·9)		231 (2·7)	250 (2·9)	
2012	253 (9·3)	301 (3·4)		277 (3·2)	301 (3·5)	
2013	111 (4·1)	113 (1·3)		106 (1·2)	113 (1·3)	
2014	47 (1·7)	23 (0·3)		21 (0·2)	22 (0·3)	

Sd: standard deviation, SMD: standardised mean difference.

[1] As the income threshold for BFP eligibility increased by 1·167 in August 2009, the monthly per capita income was divided by 1·167 for families registering with CadUnico after August 2009 in this study.

Table S5 Complete case analysis after removing individuals with missing in any matching variable. Average treatment effect on the treated (ATT)[1] of Bolsa Familia program (BFP) on leprosy cure and treatment adherence (Brazil, 2007-2014) (N=9 960).

**Outcome**	**PSM**			**IPTW**		
	**OR (95% CI)[1]**			**OR (95% CI)[1]**		
	**Overall (N=15 130)**	**PB (N=6558)**	**MB (N=8 316)**	**Overall (N=9960)**	**PB (N=4373)**	**MB (N=5587)**
**Treatment adherence[2]**						
Non-beneficiaries	1·00	1·00	1·00	1·00	1·00	1·00
Beneficiaries	1·17 (0·95-1·43)	1·29 (0·92-1·78)	1·25 (0·98-1·59)	1·34 (1·11-1·62)	1·39 (1·03-1·89)	1·37 (1·08-1·75)
**Cure**						
Non-beneficiaries	1·00	1·00	1·00	1·00	1·00	1·00
Beneficiaries	1·20 (0·94-1·52)	1·08 (0·74-1·57)	1·20 (0·91-1·59)	1·24 (1·00-1·56)	1·13 (0·80-1·59)	1·35 (1·02-1·78)

1 The average treatment effect was estimated in the propensity score matched (PSM) and inverse probability of the treatment weight (IPTW) analyses.
2OR and 95%CI estimated using logistic regression with further adjustment for income.
3Completing the minimum number of doses: ≥6 for PB or ≥12 for MB.

Table S6 Sensitivity analysis removing inconsistencies in cure and treatment adherence classification. Average treatment effect on the treated (ATT)[1] of Bolsa Familia program (BFP) on leprosy cure and treatment adherence (Brazil, 2007-2014) (N=10 794).

**Outcome**	**PSM**			**IPTW**		
	**OR (95%CI)** [1]			**OR (95%CI)** [1]		
	**Overall (N=16 284)**	**PB (N=7380)**	**MB (N=8680)**	**Overall (N=10 794)**	**PB (N=4909)**	**MB (N=5885)**
**Treatment adherence**[2]						
Non-beneficiaries	1·00	1·00	1·00	1·00	1·00	1·00
Beneficiaries	1·14 (0·91-1·43)	1·12 (0·73-1·71)	1·48 (1·16-1·90)	1·33 (1·07-1·65)	1·22 (0·85-1·73)	1·45 (1·10-1·90)
**Cure**						
Non-beneficiaries	1·00	1·00	1·00	1·00	1·00	1·00
Beneficiaries	1·08 (0·87-1·35)	1·06 (0·71-1·59)	1·40 (1·09-1·79)	1·26 (1·02-1·56)	1·13 (0·80-1·60)	1·39 (1·06-1·81)

[1] The average treatment effect was estimated in the propensity score matched (PSM) and inverse probability of the treatment weight (IPTW) analyses.
[2]OR and 95%CI estimated using logistic regression with further adjustment for income.
[3]Completing the minimum number of doses: ≥6 for PB or ≥12 for MB.

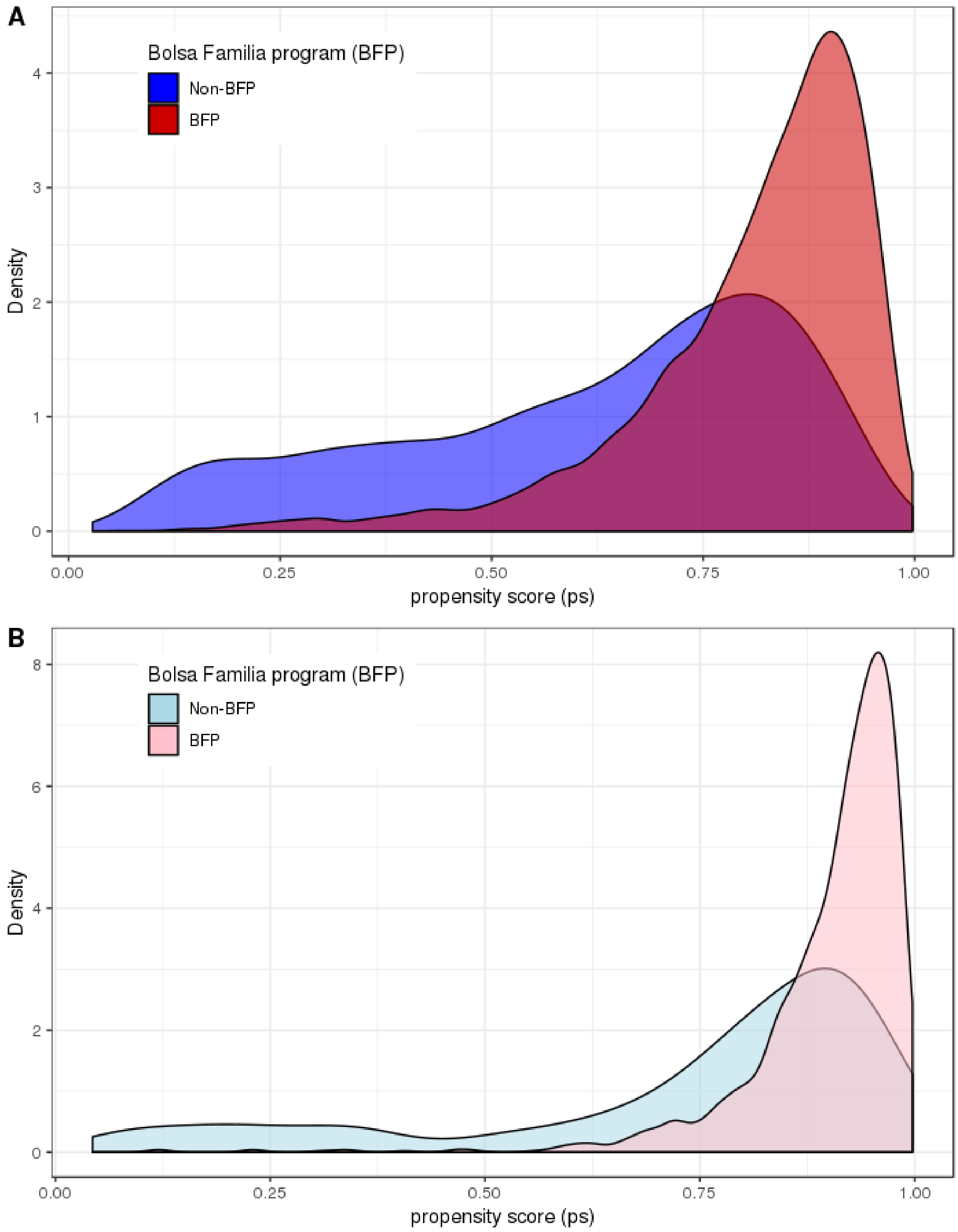

Figure S1 Probability of receiving BFP for BFP beneficiaries and non-beneficiaries given covariates for the overall population (A) and for children under 15 years (B).
